# Interventions for opioid use disorder during pregnancy: a scoping review

**DOI:** 10.3389/fpsyt.2026.1770262

**Published:** 2026-04-01

**Authors:** Amanda L. Elmore, Mirine Richey, Dewan S. Tahsin, Gabriella Hinks, William Velez-Jimenez, Allison M. Howard, Tanner Wright, Anthony Kendle, Cheryl Vamos

**Affiliations:** 1 College of Public Health, University of South Florida, Tampa, FL, United States; 2 Shimberg Health Sciences Library, University of South Florida, Tampa, FL, United States; 3 College of Medicine, University of South Florida, Tampa, FL, United States

**Keywords:** interventions, opioid use disorder, pregnancy, prenatal, scoping review

## Abstract

**Background:**

Opioid use disorder during pregnancy (OUD) continues to pose a significant public health challenge, requiring well-designed interventions. This scoping review identifies effective prenatal OUD interventions to promote the use of evidence-based approaches, such as medication for opioid use disorder (MOUD), and inform future research and clinical practice.

**Methods:**

We searched PubMed, Embase, CINAHL, Scopus, and Web of Science for interventions for OUD during pregnancy from 2013 to 2023. Exclusions were non-English studies, animal research, non-prenatal interventions, pharmacological trials, and studies outside the U.S. Data were extracted on intervention context, sample, study aims, and results. We categorized each intervention by the socioecological model (SEM) level of implementation and applied the RE-AIM framework to evaluate reach, effectiveness, adoption, implementation, and maintenance.

**Results:**

After review of 1,381 articles, a total of 31 intervention studies were included. Fourteen studies (45%) included over 75% non-Hispanic white participants. Seventeen studies (55%) were conducted at the individual level, six (19%) at the interpersonal level, and eight (26%) at the society/community level. Individual-level interventions focused on coordinated clinical care models, detoxification/tapering from (MOUD), and prenatal education. Interpersonal interventions included clinician education and group therapy. Community-level interventions targeted regional coordination of services, while society level studies examined the impact of policy change on MOUD access.

**Conclusion:**

We identified a wide range of prenatal OUD interventions with varying approaches and summarized each by SEM implementation level. While MOUD access remains crucial, community-based interventions that address broader social determinants, and societal barriers may have the greatest impact in improving maternal health outcomes.

**Systematic Review Registration:**

https://osf.io/, identifier 10.17605/OSF.IO/8ZUAB.

## Introduction

Maternal opioid use disorder (OUD) is a significant public health concern with adverse maternal and neonatal health outcomes. Maternal OUD increased by over 100% from 2010 to 2017 in the United States (US) (1). Evidence-based care for OUD during pregnancy includes a combination of behavioral therapies and medication for OUD (MOUD), such as buprenorphine or methadone (2, 3). Adherence to MOUD is associated with a decreased risk of maternal overdose and improved short and long-term health outcomes for mothers and infants (2–5). However, there remain substantial barriers to obtaining MOUD medications and long-term adherence to treatment among pregnant people (6–8).

Pregnancy provides a unique opportunity to identify and treat OUD while addressing commonly co-occurring health conditions, identifying gaps in social or financial support, and providing education to foster mother-infant wellness beyond pregnancy (4). In 2020, nearly 30% of postpartum people reported non-prescription substance use, including opioid use, and the risk increased substantially for those with stressful life events during the prenatal period (9). Therefore, a critical need remains to improve the implementation of current evidence-based practices for OUD during pregnancy and explore opportunities for improvement through public health interventions.

Intervention is a broad term to describe an activity implemented to improve health through disease prevention or reduction of disease severity (10). Interventions may be classified by their implementation setting or primary health outcome such as behavioral, social, educational, psychosocial, therapeutic, environmental, regulatory, or a combination of multiple types. Due to the breadth of the literature on various types and settings of interventions for people with OUD during pregnancy, a scoping review is necessary to outline and summarize potential interventions to improve maternal health outcomes. However, previous scoping reviews of interventions for OUD during pregnancy were limited to specific health outcomes, types of interventions, or specific implementation settings (11–14).

The purpose of this scoping literature review is to examine existing interventions for OUD during pregnancy to inform future research, policy, and clinical practice. Specifically, we aim to 1) identify studies implementing or evaluating interventions during the prenatal period for OUD, 2) describe and evaluate each intervention study, and 3) summarize and report our scoping review results. This review addresses a critical gap in the literature by providing a comprehensive, multi-level synthesis of prenatal OUD interventions across a diverse range of settings, populations, and outcomes.

## Materials and methods

### Literature search strategy

We conducted a review of the literature to identify interventions for pregnant people with OUD. Compared to a systematic review, scoping reviews are beneficial when addressing complex health issues to map key concepts and outline evidence by type and strength (15, 16). We followed the steps for conducting a scoping review by Mak and Thomas (17) and the Preferred Reporting Items for Systemic Reviews and Meta-analyses (PRISMA) guidelines (17, 18). The searches were conducted in PubMed, Embase, CINAHL, Scopus, and Web of Science. The search strategies used a combination of keywords and controlled vocabulary terms related to prenatal/pregnancy, opioid use disorder, and intervention for each database, which is consistent with the JBI Protocol (Appendix A) (19). The final protocol has been registered with the Open Science Framework (20; OSF https://doi.org/10.17605/OSF.IO/8ZUAB).

### Study selection criteria

Eligible studies implemented or evaluated interventions for pregnant people with OUD without restrictions on the type or setting of intervention. The inclusion criteria for this scoping review encompassed full-text, peer-reviewed manuscripts that were published in English between January 2013 and September 2023. We established 2013 as our start date because the standard of care recommendation from the American College of Obstetrics and Gynecologists for pregnant people with OUD was published in May 2012 (21). Our study did not have the resources to review non-English literature. Study exclusion criteria included: 1) animal studies, 2) studies that did not implement interventions during pregnancy, 3) studies strictly evaluating pharmacological interventions or clinical trials comparing mediation therapies, 4) studies conducted outside the United States, 5) studies that do not report intervention results for pregnant people or those with OUD specifically, 6) articles unavailable through institutional library of the authors, and 6) simulation intervention studies. Studies outside the US were not included because substance use research and interventions are heavily reliant upon country-specific policy. We specifically excluded pharmacological interventions given the substantial existing evidence base for MOUD efficacy and chose to focus on implementation strategies that could improve access and adherence with evidence-based pharmacotherapy.

### Data extraction, synthesis, and analysis

All records that met the search criteria were imported into Covidence for title and abstract review and full-text data extraction. We conducted a two-tiered pilot to ensure reviewer consistency for 1) study selection based on title and abstract, and 2) full-text data extraction from articles that meet search criteria. Next, each study was reviewed by two independent reviewers and a senior reviewer resolved discrepancies. To assess interrater reliability (IRR) during title and abstract screening, as well as full-text review, we utilized Covidence software, with Cohen’s kappa as the metric. The IRR (Cohen’s kappa statistic) for title and abstract screening was 0.52, and for full-text review was 0.51.

**Figure 1:**
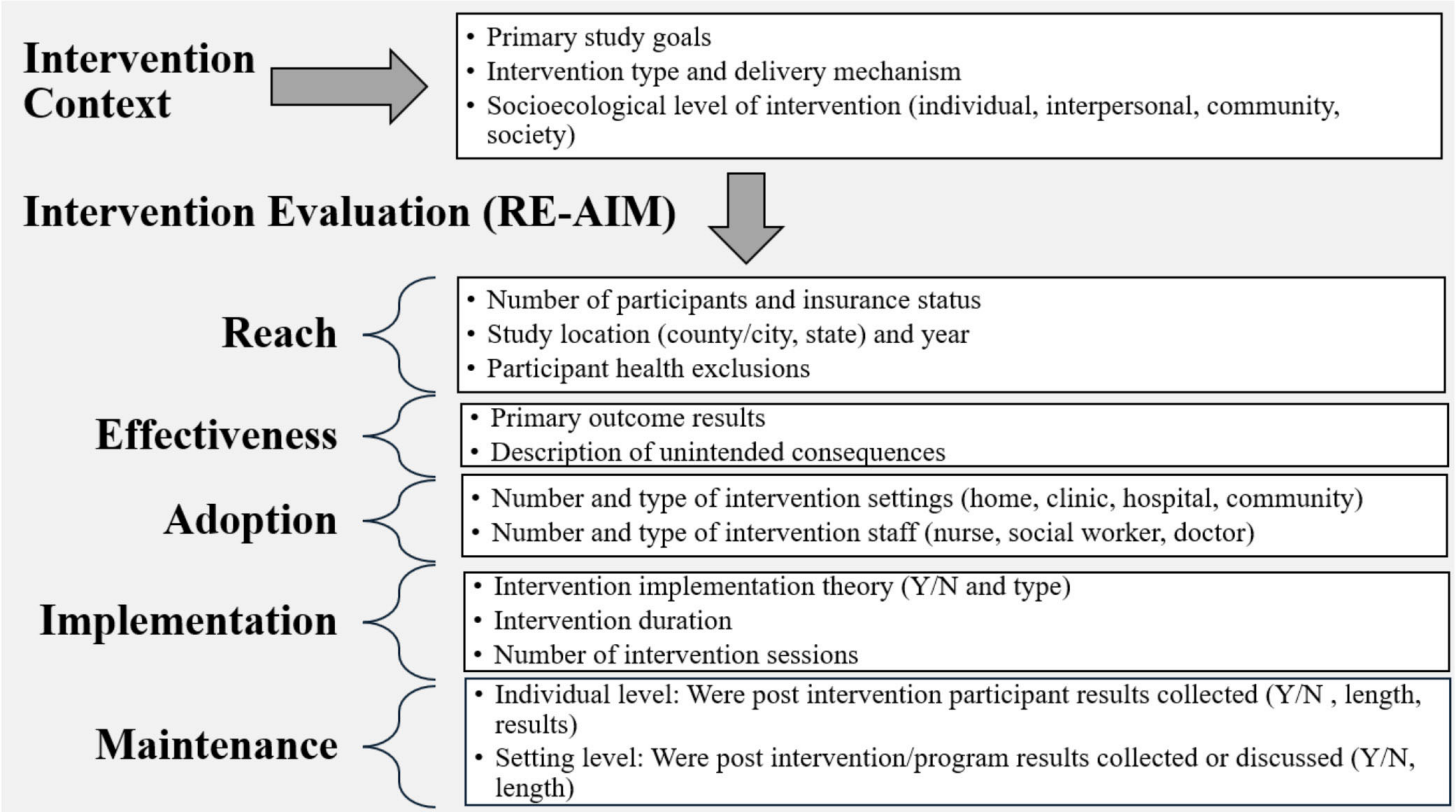
Scoping review data collection tool development outline.

Our data collection tool was developed using the RE-AIM framework to ensure our scoping review adequately described the range of interventions for pregnant people with OUD for future research and practice considerations (**Figure 1**). The RE-AIM framework was designed to improve translation of research to practice by considering aspects of design, dissemination, and implementation to facilitate equitable population-based impacts (22). To examine reach, we collected the number of participants, study location, study year, and participant health exclusions. We extracted data on participant health exclusions since many OUD intervention studies exclude pregnant people with commonly co-occurring conditions such as mood disorders, which limits the representativeness of participants (8). Effectiveness describes the impact and success of the intervention on the primary outcome, so we collected the primary study results and unintended consequences. Adoption is the intervention settings (home, clinic, hospital, etc.) and staff that deliver the intervention (nurse, social worker, doctor). Lastly, maintenance is the extent to which the intervention or program is sustained over time at the participant and setting level.

We extracted a wide range of context and evaluation variables to describe and classify the intervention studies. Context variables included the type of study, the primary intervention outcome, the type of intervention, and the level(s) of intervention implementation. To contextualize the strength of evidence, we noted the study design for each included study, categorizing designs by relative rigor: randomized controlled trials, non-randomized control trials, prospective or retrospective cohort studies, cross-sectional observational studies, and evaluations. The socioecological framework was applied to describe the primary societal level of implementation for each intervention as individual, interpersonal, community, and/or society. The socioecological model considers multiple levels of influence, which can help understand and describe the complexities of interventions aimed at improving maternal OUD outcomes (23). We provide a descriptive analysis of the eligible full texts, detailing study aims, characteristics, populations, and key findings. Additionally, we categorize the interventions according to the socioecological model, emphasizing the level of influence each intervention addresses.

## Results

### General characteristics of the studies

**Figure 2:**
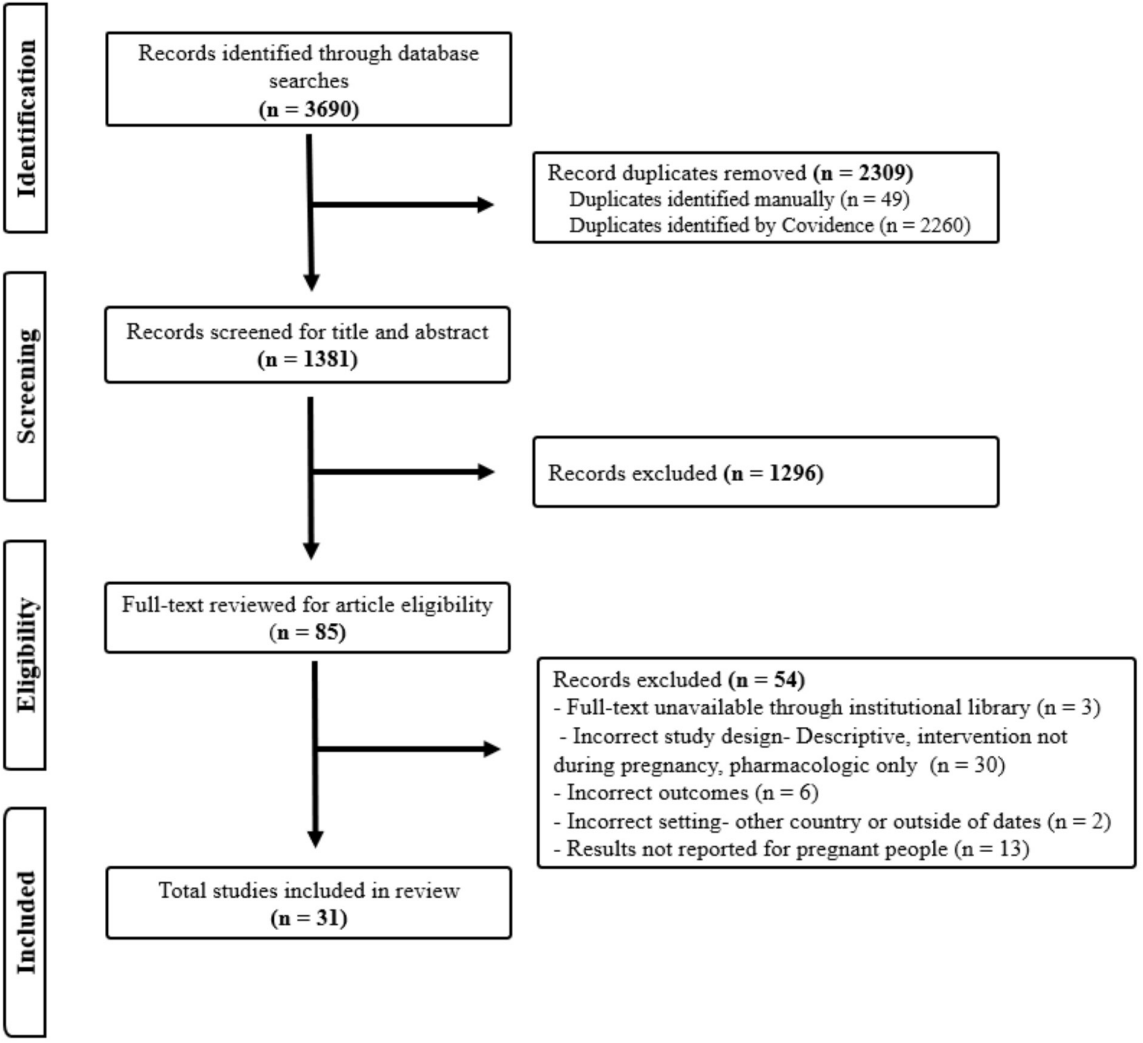
PRISMA flow diagram for selection of articles for scoping review of perinatal interventions for opioid use disorder.

**Figure 3:**
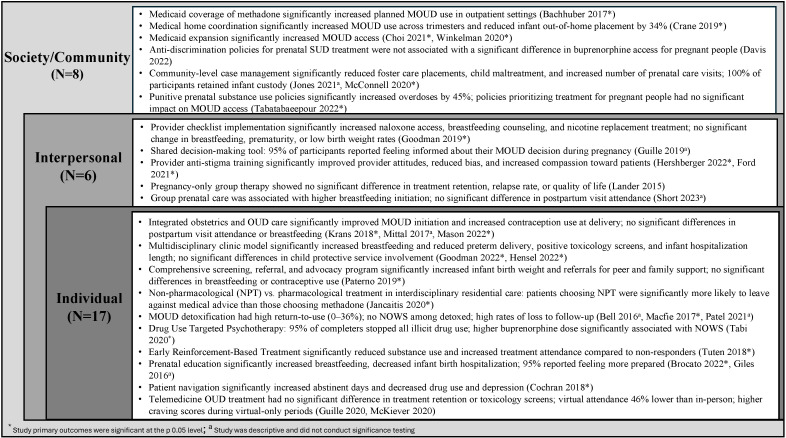
Intervention type by level of socioecological model implementation: scoping review (N = 31).

Search results from the five databases were imported into Covidence (n= 3690). After duplicate removal (n= 2,309), title and abstract screening (n=1,296), and full text-review (n=85), 31 articles were eligible for inclusion (**Figure 2**). The studies included in this review spanned the years of 2015–2023 and consisted of twelve cohort studies (retrospective & prospective), nine evaluations, seven cross-sectional studies, two randomized controlled trials, and two non-randomized control trials (**Table 1**). A total of seventeen studies (55%) were conducted at the individual level, six (19%) at the interpersonal level, and eight (26%) at the society/community level (**Figure 3**).

**Table 1:** Study intervention characteristics and outcomes by socioecological model level: scoping review 2013-2023 (N = 31).

Author/year	Study type	Study location	Sample characteristics	Intervention type	Intervention settings	MOUD treatment^a^	Primary study outcome	Secondary study outcomes
Individual socioecological model level (n=17)								
24	Cohort- retrospective or prospective	Knoxville, Tennessee	Race: NHW 88%, NHB 11% Exclusion: Not fully detoxed	Detoxification program	Home; clinic; rehabilitation center; correctional facility	Total on MOUD: 31% Buprenorphine: 31%	A total of 301 opiate-addicted pregnant patients were fully detoxified during pregnancy with no adverse fetal outcomes related to detoxification identified.	Among all participants the rate of relapse was 36% and 31% delivered an infant with NAS. The preterm delivery rate was high at 18%.
25	Cross-sectional/Observational	Alabama	Not reported	Patient education program	Clinic	Total on MOUD: 74% Methadone: 38% Buprenorphine: 23%	Higher breastfeeding rate (70% intervention vs. 24% controls, p<0.001).	Decreased length of newborn hospitalization (p<0.001) and decreased mean cost of hospitalization ($18,129 intervention vs. $34,345 control, p<0.001).
26	Cross-sectional/Observational	Pittsburgh, Pennsylvania	Race: NHW 95% Exclusion: Less than 18, non-English speaking, plans to terminate pregnancy, gestational age >25 weeks, and recent psychotic, major depressive, or manic episode	Patient Navigation Intervention	Clinic	Total on MOUD: 100% Buprenorphine: 100%	Increase in percent of past 30 days abstinent during prenatal (100%) and postnatal follow-up (96%). Significant increase in percent of days abstinent (B: 0.15, 95% CI: 0.1–0.2), decreased drug use (OR: 7.62, 95% CI: 2.8–21.0 and depression (OR: 7.70, 95% CI: 2.4–25.1).	No significant difference in overall health quality (B: 0.17, p=0.06) and substance use treatment adherence (B: 2.15, p=0.07).
27	Evaluation	Pittsburgh, Pennsylvania	Race: NHW 100%	Maternal education intervention at a Pregnancy Recovery Center	Clinic; rehabilitation center	Total on MOUD: 100% Buprenorphine: 100%	Ninety percent of women (10/11) agreed that the program helped prepare them for the immediate postpartum period while their newborns were hospitalized and understand how best to interact with newborns at risk for NAS.	No significant changes were found from baseline to post-intervention.
28	Cohort- retrospective or prospective	New England, Massachusetts	Race: NHW 99% Insurance: Medicaid 89%, private 9%, no insurance 3% Exclusion: Less than 18-year-olds, women with no prenatal care	Integrated care coordination of obstetrics/gynecology and treatment for OUD	Clinic	Total on MOUD: 96% Methadone: 18% Buprenorphine: 78%	Women receiving integrated treatment were less likely to deliver prematurely (p<0.001), less likely to have a positive toxicology screen at delivery (p<0.0001), more likely to begin prenatal care early (p<0.02), and infants had shorter hospitalizations (p<0.03).	Non-significant differences were found for rates of gestational hypertension, intrauterine growth restriction, and positive infants toxicology screen.
29	Nonrandomized control trial	South Carolina	Race: NHW 87%, NHB 9%, Hispanic 6%	Telemedicine OUD treatment	Home; clinic	Total on MOUD: 26% Buprenorphine: 26%	No significant difference on retention in treatment rate (p=0.17) or proportion of children born with NAS between the two groups (p=0.12).	No significant difference in positive drug screen at delivery (p=0.34) or 6–8 weeks postpartum (0.66) and newborn length of stay (p=0.74) between groups.
30	Cohort- retrospective or prospective	St. Louis, Missouri	Race: NHW 69%, NHB 31% Insurance: Medicaid 58%, private 42% Exclusion: Medical contraindications to breastfeeding, postpartum contraception ineligibility, no postpartum follow-up plans, no prenatal care	Multidisciplinary OUD prenatal care	Clinic	Total on MOUD: 20% Methadone: 6% Buprenorphine: 14%	Intervention patients were significantly more likely to be breastfeeding at discharge (aRR: 1.3, 95% CI: 1–1.6).	No significant difference in postpartum visit compliance (aRR: 1.0, 95% CI: 0.8–1.4) or long-term contraception use (aRR: 0.8, 95% CI: 0.4–1.8).
31	Cross-sectional/Observational	Baltimore, Maryland	Race: NHW 19%	Intensive interdisciplinary health care for substance abuse, mental health, obstetrics/gynecology, and pediatrics	Clinic; rehabilitation center	Total on MOUD: 57% Methadone: 57%	Patients that chose non-pharmacological treatment were significantly more likely to leave residential treatment against medical advice than patients who chose methadone treatment (aOR: 2.8, 95% CI: 1.2–6.2).	Leaving treatment against medical advice was significantly associated with higher age, cocaine use, patient’s rating of the importance of drug abuse and psychiatric treatment, drug interview severity rating, and family/social interviewer severity rating.
32	Cohort- retrospective or prospective	Pennsylvania	Race: NHW 94% Insurance: Medicaid 77% Exclusion: Missing prenatal and delivery data, methadone treatment	Coordinated, women-centered care	Clinic; hospital	Not reported	Intervention patients were significantly more likely to initiate buprenorphine during pregnancy vs. prior to pregnancy (p<0.01), have a higher buprenorphine dose at delivery (p=0.02), and receive a long-acting reversible contraceptive after delivery (p=0.03).	Non-significant differences were found in postpartum visit (68% vs. 53%, p=0.05) and breastfeeding at hospital discharge (49% vs. 41%, p=0.26).
33	Cohort- retrospective or prospective	Tennessee	Race: Other 7% Insurance: Medicaid 100%	MOUD vs. detoxification program	Clinic	Total on MOUD: 65%	Significant difference in opioid relapse between women on MOUD (26%), those that tapered MOUD (8%), and those that detoxed (0%) (p<0.05). Significant difference of neonatal opioid withdrawal syndrome among women on MOUD (91%), those that tapered (62%), and those that detoxed (0%) (p<0.001).	No significant difference in the use of other illicit drugs by women on MOUD (23%), those that tapered MOUD (23%), and those that detoxed (16%) (p>0.05).
34	Cohort- retrospective or prospective	Ohio	Race: NHW 100%	Multidisciplinary, coordinated care clinic	Clinic	Total on MOUD: 58% Methadone:15% Buprenorphine: 43%	Significant difference in preconception MOUD (OR: 6.5, 95% CI: 2.5-16.6), positive illicit urine drug screen at first prenatal visit (OR: 0.3, 95% CI: 0.1-0.8), and long-acting reversible contraception received at delivery (p=0.04).	No significant differences in positive urine drug screens at delivery (OR: 1.3, 95% CI: 0.4-2.9) and child protective service involvement (OR: 1.3, 95% CI: 0.5-3).
35	Evaluation	Ohio	Exclusion: Postpartum participants, no stable MOUD dosing for 4 weeks	Group psychotherapy and psychoeducation intervention	Clinic	Total on MOUD: 100%	Attendance for virtual group therapy was 46% lower than in-person and 47% lower than combined sessions (p<0.001). Significant difference in proportion that required up titration of MOUD dose during virtual visit period (p=0.02).	No significant differences were found for positive urine drug screens, emergency department visits, assaults, or overdoses. Craving scores peaked for 46% of patients during the virtual-only period but was not significant.
36	Cohort- retrospective or prospective	Boston, Massachusetts	Not reported	Collaborative care model	Clinic; hospital	Total on MOUD: 100% Buprenorphine: 100%	Buprenorphine treatment was continued until delivery in 94% (n=15) of pregnancies and the average duration of treatment was 14.5 weeks.	Among women in the program, 93% continued buprenorphine postpartum, 87% were engaged in counseling, and 10% (n=1) had a positive urine screen at delivery.
37	Cohort- retrospective or prospective	West Virginia	Race: NHW 83%, NHB 16%, Hispanic 0.1%, Other 0.6%	Outpatient MOUD tapering intervention	Clinic	Total on MOUD: 100% Buprenorphine: 100%	Fifteen percent of patients achieved opioid abstinence by delivery, 23% were still enrolled and actively tapering, and 35% were lost to follow-up, relapsed, or non-compliant. Of 14 women who achieved abstinence, 0% of babies had NOWS compared to 50% actively tapering, 51% lost to follow-up, and 38% never enrolled in program.	Mothers of infants with NOWS were significantly more likely to use tobacco (80% vs. 50%, p<0.001). Marijuana rates were significantly higher among mothers of infants without NOWS (42% vs. 28%, p=0.014).
38	Evaluation	Massachusetts	Race: NHW 95%, Hispanics 3%, Other 5%	Comprehensive screening, referral, and advocacy program	Clinic; hospital	Total on MOUD: 92% Methadone: 32% Buprenorphine: 61%	Average infant birth weight was significantly higher post-intervention (p=0.035). No significant differences were found for breast feeding initiation (p=0.180), contraceptive at hospital discharge (p=0.124), or prenatal tobacco use (p=0.485).	Significant differences post-intervention community referrals for peer support (p<0.001) and family support services (p=0.003). No significant differences in referrals for basic living needs (p=0.176) and residential treatment or detoxification programs (p=0.293).
39	Cohort- retrospective or prospective	New York	Not reported	Drug Use Targeted Therapy psychotherapy during buprenorphine maintenance	Clinic	Total on MOUD: 100% Buprenorphine: 100%	Among women that completed the program, 95% (19/20) stopped all illicit drug use, 65% (13/20) were consistently negative for cotinine, and 35% (7/20) were positive for cotinine but insisted that they were no longer inhaling tobacco. Four women discontinued the program.	Buprenorphine dose escalation was significantly associated with NOWS among the infant (p=0.009).
40	Randomized Control Trial	Baltimore, Maryland	Race: NHW 77% Exclusion: Severe medical or psychiatric conditions, comorbid conditions impacting infant outcomes, spontaneous abortion	Early Reinforcement-Based Treatment	Rehabilitation center	Total on MOUD: 92%	Interventions for Treatment Responders (TR) vs. Treatment Non-Responders (TNR) were associated with a significantly higher of number of treatment appointments attended (p=0.002) and lower rates of substance use (p<0.001). Maternal gestational age and cocaine use were negatively associated with treatment days (p< 0.001). Early treatment response, MOUD, and baseline SUD treatment were positively associated with treatment days (p<0.001).	Significant predictors of substance use at one-month post-delivery included higher level of education (p= 0.021), more advanced gestational age (p= 0.010), early TNR (p<0.0005), and baseline days of cocaine use (p=0.018).
Interpersonal socioecological model level (n=6)								
41	Evaluation	Ohio	Not reported	Trauma-informed, standardized education intervention for healthcare providers	Hospital	Not reported	Providers summary attitude scores improved significantly from 19 at baseline to 19.9 (p<0.0001) post intervention and was maintained at 20.	Providers reported changes in attitude toward people who use drugs including a decrease in anger (p=0.02), decreased blame (p<0.0001), decreased disappointment (p=0.01), and increased sympathy (p=0.002).
42	Evaluation	3 US States (NE, NH, VT)	Race: NHW 99%, NHB 0.4%, Hispanic 2%	Clinical care checklist and education program for providers	Clinic	Total on MOUD: 94% Methadone: 19% Buprenorphine: 75%	After intervention 78% of records included use of checklist. Significant increase in women with access to naloxone (p<0.001), breastfeeding counselling (p<0.01), and nicotine replacement treatment (p=0.01).	No significant change occurred in rates of prematurity, low birth weight, or breastfeeding at hospital discharge.
43	Cross-sectional/Observational	South Carolina	Race: NHW 86% Insurance: Medicaid 64%, private 36% Exclusion: Intravenous opioid use during the prior six months.	Shared decision-making tool	Clinic	Total on MOUD: 100% Methadone: 14% Buprenorphine: 78%	Ninety-five percent of participants (21/22) reported choosing to either continue MOUD (64%) or taper MOUD (36%). Reasons for tapering MOUD were concern about NOWS (100%), social service involvement (75%), stigma (75%), and having withdrawal during postpartum due to the inability to afford MOUD (63%).	Participants agreed that they were provided with sufficient information (96%) and decisional guidance (91%) to make an informed decision about MOUD during pregnancy.
44	Evaluation	Ohio	Not reported	Implicit bias training simulation game for providers	Online	Not reported	Providers had increased feelings of compassion toward the patient (p=0.001), decreased bias (p=0.0009), and decreased expectations about how difficult future encounters with the patient would be (p=0.02).	Providers had an increase in seeing the individual’s circumstances as beyond their control (p=0.0001) and an increase in effort to hide negative thoughts about patients (p=0.002).
45	Randomized Control Trial	West Virginia	Insurance: Medicaid 100% Exclusion: Alcohol use, sedative-hypnotic use disorder, untreated psychotic disorder, buprenorphine allergy, methadone use at intake; living 90-miles away from clinic, incarceration during pregnancy	Pregnancy-only tailored group therapy	Clinic	Total on MOUD: 100% Buprenorphine: 100%	No significant difference in treatment retention (p=0.90), rate of relapse (p=0.41), and change in quality of life (p=0.43).	The pregnancy-only group was more likely to rate the topics as greatly relevant compared with the treatment as usual group.
46	Cohort- retrospective or prospective	Not reported	Race: NHW 66%, Hispanic 15%, Other 32%	Group Prenatal Care	Clinic; rehabilitation center	Total on MOUD: 100%	Mothers in group prenatal care had higher rates of breast feeding initiation (80% vs. 55%), breastfeeding at delivery discharge (80% vs. 52%), and Tdap vaccine (80% vs. 71%. No difference in proportion that attended a postpartum visit (53% vs. 54%).	All group prenatal care participants (100%) reported that they would suggest the program to other mothers.
Community/society socioecological model level (n=8)								
47	Cross-sectional/Observational	30 US States (Excluded AL, CA, CT, FL, GA, HI, ID, IN, LA, MI, MN, NE, NV, NY, NC, OH, OK, RI, VT, VA, WA, WI)	Race: NHW 85%, NHB 6%, Hispanic 6%, Other 4% Insurance: Medicaid 100% Exclusion: Non-Medicaid recipients	Medicaid coverage of MOUD treatment	State level policy (N = 30)	Not reported	Admissions in states with coverage were significantly more likely to involve planned MOUD use (adjusted difference: 32.9 percentage points; 95% CI: 19.2–46.7).	The relationship persisted in intensive and non-intensive outpatient settings but was not significant in residential settings (adjusted difference: 14.3 percentage points; 95% CI: −0.7 to 29.2).
48	Cross-sectional/Observational	48 US States (Excluded FL and KY)	Exclusion: Not applicable	Medicaid expansion and prohibition of prenatal substance use policy	State level policy (N = 48)	Not reported	After Medicaid expansion, episodes receiving MOUD in states not prohibiting substance use during pregnancy increased by 8.7% (95% CI: 2.7-14.7) compared to a 5.6% increase in states prohibiting substance use during pregnancy (95% CI: −3.3-14.8).	Medicaid expansion was associated with an increase in MOUD use by 15.3% two years post-implementation in states not prohibiting versus 1.5% in states prohibiting substance use during pregnancy.
49	Cohort- retrospective or prospective	Ohio	Race: NHW 91%, NHB 6%, Other 3% Insurance: Medicaid 100% Exclusion: No prenatal care before 28 weeks gestation, non-Medicaid enrolled	Care coordination through maternal medical home model with Medicaid managed care plans	Clinic; rehabilitation center; hospital; community	MOUD: Range from 27%-69%	Increased likelihood of MOUD in trimesters one, two and three (aOR: 2.30, 4.40, 2.75, respectively, p<0.05), retention in MOUD during postpartum months 1–3 through 4-6 (aOR: 2.86, 2.40, respectively, p<0.05).	Increased behavioral health counseling during trimesters two and three (aOR: 3.75 and 2.07, respectively, p< 0.05) and lower out-of-home placement of infants (aOR: 0.66, p<0.05).
50	Evaluation	10 US States (KY, MO, TN, WV, FL, MA, MI, VA, NC, WA)	Not reported	Policy prohibiting pregnancy discrimination in SUD treatment	State level policy (N = 10)	Not reported	Non-pregnant callers were significantly more likely to obtain a buprenorphine appointment than pregnant callers (75% vs. 60% in states with a law, p<0.01; 73.1% vs. 62.3% in states without, p<0.01). No significant difference in ability to obtain methadone appointment between pregnant and non-pregnant callers between the two groups of states.	Four states had a treatment mandate, two states had mandates for publicly funded programs, and one state had mandates only for state funded programs.
51	Evaluation	Wilmington, North Carolina	Exclusion: Return to drug use, no MOUD, didn’t participate in required services	Extensive case management and care coordination	Clinic, hospital, community	Not reported	Seventy-three percent of participants reported no opiate use, 100% had full custody of their infants, 50% stayed in the program for 12–18 months, and 44% stayed for 6–12 months.	Seventy-five percent of infants were born between 36–42 weeks gestation and 61% had normal birth weights. Average hospital stay decreased from 9.6 days to 6.6 days for babies with NAS.
52	Cohort- retrospective or prospective	Oregon	Insurance: Medicaid 100% Exclusion: Diagnosis of methamphetamine use, two counties, non-Medicaid enrolled women	Community level integration of maternity care, substance use treatment, and social service linkage	Clinic, Community	Not reported	Intervention counties had a significant difference in foster care placements (-8.3%, p<0.001), child maltreatment (-7.2%, p<0.001), and number of prenatal care visits (1.1%, p<0.001).	No significant differences found between counties for preterm birth, low birth weight, or high-needs care for infants (p>0.05).
53	Evaluation	47 US States (Excluded ID, NM, RI)	Insurance: Private 100%	State-level prenatal substance use policies (PSUP)	State level policy (N = 47)	Total on MOUD: 12% Methadone: 0.6% Buprenorphine: 11% Naltrexone: 0.4%	After PSUP’s funding targeted SUD treatment programs, opioid overdose decreased by 45% and use of MOUD increased by 11%. PSUPs that prioritized SUD treatment for pregnant people did not significantly change MOUD treatment access.	Punitive PSUPs decreased psychosocial services for SUD by 12% and methadone treatment by 30%. Criminalizing PSUPs significantly increased opioid overdoses by 45% (p<0.01).
54	Cross-sectional/Observational	49 US states (Excluded FL)	Race: NHW 68%, NHB 14%, Hispanic 5%, American Indian and Alaska Native 3%, Other 11%	Medicaid expansion and type of treatment referrals	State level policy (N = 49)		MOUD receipt significantly differed by referral agency with 26% for criminal justice agencies, 59% for individual, and 51% for other referrals (p<0.001).	Pregnant women referred by criminal justice agencies were significantly more likely to have received medications for OUD in states that expanded Medicaid (p=0.04).

aOR, adjusted odds ratio; aRR, adjusted relative risk; B=beta; CI, confidence interval; MAT, Medication assisted treatment; MOUD, Medication for opioid use disorder; NAS, Neonatal Abstinence Syndrome; NHB, non-Hispanic Black; NHW, non-Hispanic White; NOWS, Neonatal Opioid Withdrawal Syndrome; OAT, opioid agonist therapy; OUD, Opioid Use Disorder; OR, odds ratio; PSUP, prenatal substance use policies; SUD, substance use disorder.

### Participant characteristics

The average number of pregnant study participants was 8,675 with a minimum of 13 and maximum of 131,838. Forty-five percent of studies (n=14) had over 75% non-Hispanic white participants (**Table 1**). Six studies reported less than 100% of non-Hispanic white participants but did not report the race/ethnicity of the remaining participants. Only 10 (32%) studies reported participants with a race/ethnicity other than non-Hispanic white. Among the studies that reported insurance status (n=11), five studies (45%) included only Medicaid recipients, and one study included only privately insured individuals (8%). Four studies did not report sample race/ethnicity or insurance status of the participants (25, 36, 41, 50). Twenty-four (77%) of the reviewed studies included one state, six (19%) included multiple states, and one study did not report their intervention location (**Table 1**) (46).

### Intervention characteristics and outcomes by socioecological level: individual

Seventeen studies (55%) included interventions implemented at the individual level (**Table 2**; **Figure 3**). The average number of pregnant participants for individual level studies was 151 with a range of 13-783. Among studies that reported participant characteristics (n=13), 69% (n=9) included over 80% non-Hispanic white participants and one (8%) study included only Medicaid recipients (33). Ninety-four percent (n=16) of studies conducted at the individual level reported a proportion of intervention participants were taking MOUD (range: 20% to 100%), which included methadone or buprenorphine (**Table 1**). Six studies reported that 100% of the participants were taking MOUD; of which, 83% (n=5) included participants prescribed buprenorphine only. No studies reported only methadone use among participants.

**Table 2:** Study intervention characteristics and outcomes by socioecological model level: scoping review 2013-2023 (N = 31).

Author/year	Intervention type	Intervention settings	MOUD treatment^a^	Primary study outcome	Secondary study outcomes
Individual socioecological model level (n=17)					
24	Detoxification program	Home; clinic; rehabilitation center; correctional facility	Total on MOUD: 31% Buprenorphine: 31%	A total of 301 opiate-addicted pregnant patients were fully detoxified during pregnancy with no adverse fetal outcomes related to detoxification identified.	Among all participants the rate of relapse was 36% and 31% delivered an infant with NAS. The preterm delivery rate was high at 18%.
25	Patient education program	Clinic	Total on MOUD: 74% Methadone: 38% Buprenorphine: 23%	Higher breastfeeding rate (70% intervention vs. 24% controls, p<0.001).	Decreased length of newborn hospitalization (p<0.001) and decreased mean cost of hospitalization ($18,129 intervention vs. $34,345 control, p<0.001).
26	Patient Navigation Intervention	Clinic	Total on MOUD: 100% Buprenorphine: 100%	Increase in percent of past 30 days abstinent during prenatal (100%) and postnatal follow-up (96%). Significant increase in percent of days abstinent (B: 0.15, 95% CI: 0.1–0.2), decreased drug use (OR: 7.62, 95% CI: 2.8–21.0 and depression (OR: 7.70, 95% CI: 2.4–25.1).	No significant difference in overall health quality (B: 0.17, p=0.06) and substance use treatment adherence (B: 2.15, p=0.07).
27	Maternal education intervention at a Pregnancy Recovery Center	Clinic; rehabilitation center	Total on MOUD: 100% Buprenorphine: 100%	Ninety percent of women (10/11) agreed that the program helped prepare them for the immediate postpartum period while their newborns were hospitalized and understand how best to interact with newborns at risk for NAS.	No significant changes were found from baseline to post-intervention.
28	Integrated care coordination of obstetrics/gynecology and treatment for OUD	Clinic	Total on MOUD: 96% Methadone: 18% Buprenorphine: 78%	Women receiving integrated treatment were less likely to deliver prematurely (p<0.001), less likely to have a positive toxicology screen at delivery (p<0.0001), more likely to begin prenatal care early (p<0.02), and infants had shorter hospitalizations (p<0.03).	Non-significant differences were found for rates of gestational hypertension, intrauterine growth restriction, and positive infants toxicology screen.
29	Telemedicine OUD treatment	Home; clinic	Total on MOUD: 26% Buprenorphine: 26%	No significant difference on retention in treatment rate (p=0.17) or proportion of children born with NAS between the two groups (p=0.12).	No significant difference in positive drug screen at delivery (p=0.34) or 6–8 weeks postpartum (0.66) and newborn length of stay (p=0.74) between groups.
30	Multidisciplinary OUD prenatal care	Clinic	Total on MOUD: 20% Methadone: 6% Buprenorphine: 14%	Intervention patients were significantly more likely to be breastfeeding at discharge (aRR: 1.3, 95% CI: 1–1.6).	No significant difference in postpartum visit compliance (aRR: 1.0, 95% CI: 0.8–1.4) or long-term contraception use (aRR: 0.8, 95% CI: 0.4–1.8).
31	Intensive interdisciplinary health care for substance abuse, mental health, obstetrics/gynecology, and pediatrics	Clinic; rehabilitation center	Total on MOUD: 57% Methadone: 57%	Patients that chose non-pharmacological treatment were significantly more likely to leave residential treatment against medical advice than patients who chose methadone treatment (aOR: 2.8, 95% CI: 1.2–6.2).	Leaving treatment against medical advice was significantly associated with higher age, cocaine use, patient’s rating of the importance of drug abuse and psychiatric treatment, drug interview severity rating, and family/social interviewer severity rating.
32	Coordinated, women-centered care	Clinic; hospital	Not reported	Intervention patients were significantly more likely to initiate buprenorphine during pregnancy vs. prior to pregnancy (p<0.01), have a higher buprenorphine dose at delivery (p=0.02), and receive a long-acting reversible contraceptive after delivery (p=0.03).	Non-significant differences were found in postpartum visit (68% vs. 53%, p=0.05) and breastfeeding at hospital discharge (49% vs. 41%, p=0.26).
33	MOUD vs. detoxification program	Clinic	Total on MOUD: 65%	Significant difference in opioid relapse between women on MOUD (26%), those that tapered MOUD (8%), and those that detoxed (0%) (p<0.05). Significant difference of neonatal opioid withdrawal syndrome among women on MOUD (91%), those that tapered (62%), and those that detoxed (0%) (p<0.001).	No significant difference in the use of other illicit drugs by women on MOUD (23%), those that tapered MOUD (23%), and those that detoxed (16%) (p>0.05).
34	Multidisciplinary, coordinated care clinic	Clinic	Total on MOUD: 58% Methadone:15% Buprenorphine: 43%	Significant difference in preconception MOUD (OR: 6.5, 95% CI: 2.5-16.6), positive illicit urine drug screen at first prenatal visit (OR: 0.3, 95% CI: 0.1-0.8), and long-acting reversible contraception received at delivery (p=0.04).	No significant differences in positive urine drug screens at delivery (OR: 1.3, 95% CI: 0.4-2.9) and child protective service involvement (OR: 1.3, 95% CI: 0.5-3).
35	Group psychotherapy and psychoeducation intervention	Clinic	Total on MOUD: 100%	Attendance for virtual group therapy was 46% lower than in-person and 47% lower than combined sessions (p<0.001). Significant difference in proportion that required up titration of MOUD dose during virtual visit period (p=0.02).	No significant differences were found for positive urine drug screens, emergency department visits, assaults, or overdoses. Craving scores peaked for 46% of patients during the virtual-only period but was not significant.
36	Collaborative care model	Clinic; hospital	Total on MOUD: 100% Buprenorphine: 100%	Buprenorphine treatment was continued until delivery in 94% (n=15) of pregnancies and the average duration of treatment was 14.5 weeks.	Among women in the program, 93% continued buprenorphine postpartum, 87% were engaged in counseling, and 10% (n=1) had a positive urine screen at delivery.
37	Outpatient MOUD tapering intervention	Clinic	Total on MOUD: 100% Buprenorphine: 100%	Fifteen percent of patients achieved opioid abstinence by delivery, 23% were still enrolled and actively tapering, and 35% were lost to follow-up, relapsed, or non-compliant. Of 14 women who achieved abstinence, 0% of babies had NOWS compared to 50% actively tapering, 51% lost to follow-up, and 38% never enrolled in program.	Mothers of infants with NOWS were significantly more likely to use tobacco (80% vs. 50%, p<0.001). Marijuana rates were significantly higher among mothers of infants without NOWS (42% vs. 28%, p=0.014).
38	Comprehensive screening, referral, and advocacy program	Clinic; hospital	Total on MOUD: 92% Methadone: 32% Buprenorphine: 61%	Average infant birth weight was significantly higher post-intervention (p=0.035). No significant differences were found for breast feeding initiation (p=0.180), contraceptive at hospital discharge (p=0.124), or prenatal tobacco use (p=0.485).	Significant differences post-intervention community referrals for peer support (p<0.001) and family support services (p=0.003). No significant differences in referrals for basic living needs (p=0.176) and residential treatment or detoxification programs (p=0.293).
39	Drug Use Targeted Therapy psychotherapy during buprenorphine maintenance	Clinic	Total on MOUD: 100% Buprenorphine: 100%	Among women that completed the program, 95% (19/20) stopped all illicit drug use, 65% (13/20) were consistently negative for cotinine, and 35% (7/20) were positive for cotinine but insisted that they were no longer inhaling tobacco. Four women discontinued the program.	Buprenorphine dose escalation was significantly associated with NOWS among the infant (p=0.009).
40	Early Reinforcement-Based Treatment	Rehabilitation center	Total on MOUD: 92%	Interventions for Treatment Responders (TR) vs. Treatment Non-Responders (TNR) were associated with a significantly higher of number of treatment appointments attended (p=0.002) and lower rates of substance use (p<0.001). Maternal gestational age and cocaine use were negatively associated with treatment days (p< 0.001). Early treatment response, MOUD, and baseline SUD treatment were positively associated with treatment days (p<0.001).	Significant predictors of substance use at one-month post-delivery included higher level of education (p= 0.021), more advanced gestational age (p= 0.010), early TNR (p<0.0005), and baseline days of cocaine use (p=0.018).
Interpersonal socioecological model level (n=6)					
41	Trauma-informed, standardized education intervention for healthcare providers	Hospital	Not reported	Providers summary attitude scores improved significantly from 19 at baseline to 19.9 (p<0.0001) post intervention and was maintained at 20.	Providers reported changes in attitude toward people who use drugs including a decrease in anger (p=0.02), decreased blame (p<0.0001), decreased disappointment (p=0.01), and increased sympathy (p=0.002).
42	Clinical care checklist and education program for providers	Clinic	Total on MOUD: 94% Methadone: 19% Buprenorphine: 75%	After intervention 78% of records included use of checklist. Significant increase in women with access to naloxone (p<0.001), breastfeeding counselling (p<0.01), and nicotine replacement treatment (p=0.01).	No significant change occurred in rates of prematurity, low birth weight, or breastfeeding at hospital discharge.
43	Shared decision-making tool	Clinic	Total on MOUD: 100% Methadone: 14% Buprenorphine: 78%	Ninety-five percent of participants (21/22) reported choosing to either continue MOUD (64%) or taper MOUD (36%). Reasons for tapering MOUD were concern about NOWS (100%), social service involvement (75%), stigma (75%), and having withdrawal during postpartum due to the inability to afford MOUD (63%).	Participants agreed that they were provided with sufficient information (96%) and decisional guidance (91%) to make an informed decision about MOUD during pregnancy.
44	Implicit bias training simulation game for providers	Online	Not reported	Providers had increased feelings of compassion toward the patient (p=0.001), decreased bias (p=0.0009), and decreased expectations about how difficult future encounters with the patient would be (p=0.02).	Providers had an increase in seeing the individual’s circumstances as beyond their control (p=0.0001) and an increase in effort to hide negative thoughts about patients (p=0.002).
45	Pregnancy-only tailored group therapy	Clinic	Total on MOUD: 100% Buprenorphine: 100%	No significant difference in treatment retention (p=0.90), rate of relapse (p=0.41), and change in quality of life (p=0.43).	The pregnancy-only group was more likely to rate the topics as greatly relevant compared with the treatment as usual group.
46	Group Prenatal Care	Clinic; rehabilitation center	Total on MOUD: 100%	Mothers in group prenatal care had higher rates of breastfeeding initiation (80% vs. 55%), breastfeeding at delivery discharge (80% vs. 52%), and Tdap vaccine (80% vs. 71%. No difference in proportion that attended a postpartum visit (53% vs. 54%).	All group prenatal care participants (100%) reported that they would suggest the program to other mothers.
Community/society socioecological model level (n=8)					
47	Medicaid coverage of MOUD treatment	State level policy (N = 30)	Not reported	Admissions in states with coverage were significantly more likely to involve planned MOUD use (adjusted difference: 32.9 percentage points; 95% CI: 19.2–46.7).	The relationship persisted in intensive and non-intensive outpatient settings but was not significant in residential settings (adjusted difference: 14.3 percentage points; 95% CI: −0.7 to 29.2).
48	Medicaid expansion and prohibition of prenatal substance use policy	State level policy (N = 48)	Not reported	After Medicaid expansion, episodes receiving MOUD in states not prohibiting substance use during pregnancy increased by 8.7% (95% CI: 2.7-14.7) compared to a 5.6% increase in states prohibiting substance use during pregnancy (95% CI: −3.3-14.8).	Medicaid expansion was associated with an increase in MOUD use by 15.3% two years post-implementation in states not prohibiting versus 1.5% in states prohibiting substance use during pregnancy.
49	Care coordination through maternal medical home model with Medicaid managed care plans	Clinic; rehabilitation center; hospital; community	MOUD: Range from 27%-69%	Increased likelihood of MOUD in trimesters one, two and three (aOR: 2.30, 4.40, 2.75, respectively, p<0.05), retention in MOUD during postpartum months 1–3 through 4-6 (aOR: 2.86, 2.40, respectively, p<0.05).	Increased behavioral health counseling during trimesters two and three (aOR: 3.75 and 2.07, respectively, p< 0.05) and lower out-of-home placement of infants (aOR: 0.66, p<0.05).
50	Policy prohibiting pregnancy discrimination in SUD treatment	State level policy (N = 10)	Not reported	Non-pregnant callers were significantly more likely to obtain a buprenorphine appointment than pregnant callers (75% vs. 60% in states with a law, p<0.01; 73.1% vs. 62.3% in states without, p<0.01). No significant difference in ability to obtain methadone appointment between pregnant and non-pregnant callers between the two groups of states.	Four states had a treatment mandate, two states had mandates for publicly funded programs, and one state had mandates only for state funded programs.
51	Extensive case management and care coordination	Clinic, hospital, community	Not reported	Seventy-three percent of participants reported no opiate use, 100% had full custody of their infants, 50% stayed in the program for 12–18 months, and 44% stayed for 6–12 months.	Seventy-five percent of infants were born between 36–42 weeks gestation and 61% had normal birth weights. Average hospital stay decreased from 9.6 days to 6.6 days for babies with NAS.
52	Community level integration of maternity care, substance use treatment, and social service linkage	Clinic, Community	Not reported	Intervention counties had a significant difference in foster care placements (-8.3%, p<0.001), child maltreatment (-7.2%, p<0.001), and number of prenatal care visits (1.1%, p<0.001).	No significant differences found between counties for preterm birth, low birth weight, or high-needs care for infants (p>0.05).
53	State-level prenatal substance use policies (PSUP)	State level policy (N = 47)	Total on MOUD: 12% Methadone: 0.6% Buprenorphine: 11% Naltrexone: 0.4%	After PSUP’s funding targeted SUD treatment programs, opioid overdose decreased by 45% and use of MOUD increased by 11%. PSUPs that prioritized SUD treatment for pregnant people did not significantly change MOUD treatment access.	Punitive PSUPs decreased psychosocial services for SUD by 12% and methadone treatment by 30%. Criminalizing PSUPs significantly increased opioid overdoses by 45% (p<0.01).
54	Medicaid expansion and type of treatment referrals	State level policy (N = 49)		MOUD receipt significantly differed by referral agency with 26% for criminal justice agencies, 59% for individual, and 51% for other referrals (p<0.001).	Pregnant women referred by criminal justice agencies were significantly more likely to have received medications for OUD in states that expanded Medicaid (p=0.04).

a. participant treatment with medication for opioid use disorder (MOUD) during intervention study.

aOR, adjusted odds ratio; aRR, adjusted relative risk; B=beta; CI, confidence interval; NAS, Neonatal Abstinence Syndrome; NOWS, Neonatal Opioid Withdrawal Syndrome; OR, odds ratio; PSUP, prenatal substance use policies; SUD, substance use disorder.

The main types of individual level interventions included: coordinated clinical care models (n=7, 44%), detoxification/tapering from MOUD (n= 3, 19%), prenatal education (n=2, 13%), and telemedicine OUD treatment (n=2, 13%) (**Table 1**, **Figure 3**). The majority of individual level interventions were conducted at one clinic or rehabilitation center (n=13, 76%), but the number of settings ranged from one to four. Only two articles clearly reported the number of intervention sessions for participants as 14 total office visits and 2 intervention sessions (25, 26). The length of intervention implementation ranged from eight weeks to four years, but six (35%) studies did not report the length of the intervention (24, 25, 30, 31, 34, 50). Only five (29%) studies reported using an implementation theory (26, 27, 37–39).

A wide range of maternal and infant health outcomes were included for individual level intervention study outcomes. The most commonly reported outcomes were positive maternal toxicology screening or opioid return to use rate, illicit substance use, Neonatal Opioid Withdrawal Syndrome (NOWS), adherence to MOUD or treatment regimen, and breastfeeding (**Table 1**). The most common form of individual level intervention included coordination of prenatal care and OUD treatment, which showed positive results for some outcomes. Krans et al. (32) and Mason et al. (34) found that participants were significantly more likely to begin MOUD early in pregnancy or during preconception. A few coordinated care studies had conflicting results including Goodman et al. (28) found that participants were significantly less likely to have a positive toxicology screen at delivery, but Mason et al. (34) reported no significant change in maternal toxicology screens. Also, Hensel et al. (30) found that participants were significantly more likely to breastfeed, but Krans et al. (32) and Paterno et al. (38) found no significant change in breastfeeding. Two of the coordinated clinical care models examined postpartum visit adherence and found no significant changes (30, 32). Two studies reported intervention impacts on child protective service involvement and neither found significant differences post-intervention (28, 34).

Three individual level interventions included detoxing participants from MOUD, despite MOUD being the standard of care for perinatal OUD (**Table 1**) (24, 33, 37). A MOUD detoxification intervention with 301 participants in North Carolina reported no adverse infant health outcomes, but a 36% (n= 107) or return to use, or relapse, rate among participants at delivery (24). One study compared return to use among pregnant people that detoxed or remained on MOUD in Tennessee and reported those that remained on MOUD were significantly more likely to return to use than those that tapered or detoxed (p<0.05) (33). Lastly, Patel et al. (37) examined an outpatient detoxification program in West Virginia and found that only 15% of participants fully detoxed by delivery and 35% were lost to follow-up, returned to use, or were non-compliant. The NOWS rate for participants that fully detoxed and had no signs of return to use was 0% in all three studies.

Two studies examined the effectiveness of OUD telemedicine treatment but did not report positive results (**Table 1**). Guille et al. (29) found that OUD telemedicine treatment provided in South Carolina during 2017 had no significant impacts on treatment retention or positive maternal toxicology at delivery. The study by McKiever et al. (35) evaluated the change to telemedicine due to the COVID-19 pandemic and reported a 46% lower attendance rate at virtual visits than in-person, higher craving scores during virtual only study periods, and no significant differences in positive maternal toxicology screens. However, the change to telemedicine due to the pandemic was not associated with a higher rate of overdose (35). Four (24%) individual level intervention studies reported unintended consequences including overdose and return to use (24, 26, 33, 35).

### Intervention characteristics and outcomes by socioecological level: interpersonal

Six studies (19%) included interventions implemented at the interpersonal level (**Table 2**; **Figure 3**). Four interpersonal level interventions directly included pregnant people, and the average number of participants was 90. All interpersonal level studies with pregnant participants included over 65% non-Hispanic white participants and had over 90% of participants taking MOUD with buprenorphine the most common form (**Table 1**). The main types of interpersonal level interventions were healthcare provider anti-stigma education programs (n=2) (41, 44), clinical care improvement initiatives (n=2) (28, 43), and group prenatal therapy/care (n=2) (**Table 1**, **Figure 3**) (45, 46). Four studies (67%) reported utilizing an implementation theory (28, 36, 44, 50), two of which reported using the Plan, Do, Study, Act cycles (28, 41).

Two interpersonal level articles included healthcare providers as their intervention participants. Ford et al. (41) conducted a trauma-informed standardized educational intervention in 39 Ohio hospitals among 1,873 healthcare workers, including medical doctors (7%), nurse practitioners (5%), neonatal nurses (76%), social workers (2%), and other health professionals (9%). Hershberger et al. (44) evaluated a virtual training simulation game to reduce healthcare provider (n=52) bias against pregnant people with OUD but did not report participant demographics specifically for the scenario of interest. Both articles found significant changes in levels of anger, bias, disappointment, sympathy, and blame towards pregnant patients with OUD (**Table 1**).

Clinical care improvement interventions included implementation of a new clinical tool, provider education, and group prenatal care or therapy. Goodman et al. (28) evaluated the implementation of a provider checklist and found an increase in women with access to naloxone (p<0.001), breastfeeding counselling (p<0.01), and nicotine replacement treatment (p=0.01) (**Table 1**). No significant differences were found in rates of low birth weight or breastfeeding (28). Guille et al. (43) implemented a shared decision-making tool to ensure patients make informed treatment decisions that reflect their individual preferences and values. Over 90% of participants reported feeling informed about their decision about MOUD during pregnancy (**Table 1**) (43). Neither group therapy or prenatal care interventions found a significant difference in treatment retention or attendance (**Table 1**) (45, 46). However, both reported positive feedback from that participants they would suggest the program to other mothers or found the topics more relevant than usual treatment groups (45, 46). Lander et al. (45) conducted a pregnancy-only group therapy intervention but found no significant differences in the return to use rate or quality of life. Short et al. (46) implemented a group prenatal care model and found higher rates of breastfeeding initiation and at delivery discharge.

### Intervention characteristics and outcomes by socioecological level: community or society

Eight articles (26%) included interventions primarily implemented at the community or society level (**Table 2**; **Figure 3**). The community level interventions (n=3, 38%) included an average of 894 pregnant participants (range: 53-1,531). Both community studies that reported participant insurance status included only Medicaid recipients (**Table 1**). The society level intervention studies (n=5, 63%) included a range of 10 to 49 states and an average of 59,320 pregnant people (range: 3,354-131,838) (**Table 1**). Three society level studies reported participant characteristics (47, 53, 54); of which, all included a majority of non-Hispanic white participants. One society level intervention reported the proportion of participants on MOUD as 12% with 11% prescribed buprenorphine (**Table 1**) (53).

Studies with community level interventions consisted of coordination of health and social services for pregnant people with OUD at the county or city level and examined outcomes such as retention in treatment, MOUD use, and child removal. Crane et al. (49) found that care coordination through a medical home model with Medicaid managed plans in Ohio increased prenatal and postpartum MOUD use (p<0.05) and resulted in a 34% lower out of home placement for infants (**Table 1**). Jones et al. (51) evaluated community case management and reported 100% of participants retained custody of their infants. McConnell et al. (52) assessed county level coordination in Oregon and found that intervention counties had significantly lower foster care placements (p<0.001) and child maltreatment reports (p<0.001). One study reported surveying previous participants to assess long-term health outcomes, and the findings were widely positive (51). Two of the community level studies reported plans to continue the interventions long-term (51, 52).

All studies at the society level (n=5) examined the impact of state level policy change on access to MOUD among pregnant people (**Table 1**). The articles examined a variety of state policies including Medicaid expansion (n=2) (48, 54), prenatal substance use policies (PSUPs) (n=1) (53). Medicaid coverage of methadone (n=1), (47) and anti-discrimination for prenatal OUD treatment (n=1) (**Table 1**) (50). Winkelman et al. (54) examined Medicaid expansion by type of treatment referral and found that pregnant people were least likely to be referred by criminal justice agencies overall. However, those referred by criminal justice agencies were significantly more likely to receive MOUD in states that expanded Medicaid (p<0.05) (54). Choi et al. (48) assessed the interaction of Medicaid expansion and punitive PSUPs and reported that Medicaid expansion was associated with an increase in MOUD use by 15% in states without punitive PSUPs compared to 2% in states with PSUPs. Tabatabaeepour et al. (53) examined types of PSUPs and reported that PSUPs that prioritized SUD treatment for pregnant people did not significantly change MOUD access and criminalizing PSUPs significantly increased opioid overdoses by 45% (p<0.01). Davis et al. (50) also found no significant difference in ability to obtain an appointment for methadone in states with PSUPs that prioritized SUD treatment for pregnant people.

## Discussion

We conducted a novel scoping review to identify interventions for pregnant people with OUD and utilized a broad inclusion criterion to capture a variety of intervention approaches, maternal health outcomes of interest, and multi-level implementation settings. Our review assessed nearly 1400 studies from 2013 to 2023, and only 31 studies were ultimately included in our final review. The majority of studies were retrospective cohorts or observational designs, thus conclusions about intervention effectiveness must be interpreted in the context of study design limitations. We examined the interventions using the RE-AIM framework and classified each according to the socioecological model, finding that the majority targeted individual-level factors, followed by interpersonal, community, and societal levels. Our findings emphasize the strengths and limitations of current prenatal OUD interventions, revealing critical gaps in participant representativeness, intervention effectiveness and sustainability, and the paucity of prenatal focused interventions for the seven-year article search timeframe. In this review, we found that the majority of studies had low participant diversity, or reach, as defined by our RE-AIM framework (**Figure 1**). Over half of the studies had more than 75% of participants identified as non-Hispanic white. In stark contrast with research guidelines and ethics, seven studies with less than 100% of non-Hispanic white participants failed to report the race/ethnicity of the remaining participants. A recent scoping review by Schiff, Work, Foley, et al. (55) used the Public Health Critical Race Framework to assess the reporting and inclusion of race/ethnicity data for research on maternal-infant dyads affected by OUD (55). Similar to our findings, the study concluded that literature from 2000 to 2020 poorly reported data on race/ethnicity and found that few studies focused on examining race/ethnic disparities (55). Racial inequities in maternal OUD treatment are evident, with non-White pregnant individuals being less likely to initiate and continue treatment, including lower prescribing rates and access to MOUD (56–59). Thus, the underrepresentation and underreporting of racial and ethnic minorities in perinatal OUD research raises concerns about the generalizability of current intervention findings and highlights the need for more inclusive research to improve outcomes for all pregnant individuals with OUD.

Our review demonstrated significant gaps in reporting on the adoption, implementation, and maintenance of interventions, as highlighted by the RE-AIM framework. In terms of adoption, the number and type of intervention settings and staff reported were often vague or not provided in the articles. Eleven (35%) of the reviewed studies indicated use of an implementation theory for their intervention design or evaluation with wide variation in the type. The intervention duration and number of sessions was often not reported for implementation. Regarding maintenance, there was a noticeable deficiency in long-term follow-up data. While one study surveyed former participants for long-term health outcomes, and two community-level studies indicated plans for continued intervention, the absence of systematic evaluations limits future researchers’ ability to identify interventions with proven long-term effectiveness. Collectively, these gaps in the literature highlight the need for more thorough reporting to inform the adoption, implementation, and sustainability of effective interventions for prenatal OUD.

Our review identified notable differences in intervention effectiveness depending upon service integration, intensity, timing, and consistency with standards of care. When examining intervention effectiveness by socioecological levels, community-based approaches (26% of included studies), consistently demonstrated more robust results than individual-level interventions (55% of included studies). Effective interventions shared three key characteristics: 1) integration of multiple evidence-based clinical service types, 2) addressing social determinants of health in addition to clinical care, and 3) sustaining engagement with mothers throughout the perinatal period. The key difference was how “care coordination” was operationalized for community and individual level interventions. Individual-level coordination (prenatal care, MOUD management, education, telemedicine) showed mixed results with limited reach (76% single-clinic), while community-level coordination integrating clinical care with social services consistently achieved positive outcomes, including significant reductions in child removals (49, 52). Adherence to evidence-based standards is essential with all three MOUD detoxification studies showing high return to use rates and low program completion. These findings underscore the risks of detoxification during pregnancy and emphasize the importance of MOUD as the standard of care. However, patient desire to discontinue MOUD due to concerns for NOWS, social service involvement, and clinician stigma highlights the importance of shared decision-making tools and anti-stigma training, both of which showed positive results (8, 43).

While the predominance of observational designs limits causal conclusions, consistent patterns across independent studies support guidance for policy and practice. Interventions with the most consistent positive findings, community-level care coordination and integrated OB-OUD models, suggest these approaches may be more effective for addressing the full spectrum of needs faced by pregnant individuals with OUD. This finding aligns with the complexity of perinatal OUD, which extends beyond access to treatment to include comorbidities such as infectious diseases and mental health disorders (23, 60), as well as housing instability, food insecurity, and involvement with law enforcement and child welfare services (61, 62). These interconnected barriers explain why individual-level clinical interventions showed mixed results while community-level approaches consistently demonstrated positive outcomes. Interventions that demonstrated promise but require further evaluation include anti-stigma provider training and group prenatal care. Detoxification and tapering approaches demonstrated consistent harm across three independent studies and are not supported by the current evidence base.

Policy changes that improve access to MOUD and other supportive services, especially for Medicaid recipients and those in states with punitive PSUPs, are essential for improving outcomes for families affected by OUD (63). Notably, criminalizing PSUPs were associated with a 45% increase in opioid overdoses, highlighting that punitive approaches not only fail to improve outcomes but actively cause harm (53). Punitive PSUPs disproportionately affect low-income and minority pregnant individuals who already face the greatest barriers to care, making reform an urgent maternal and child health equity priority (64). Our review of societal-level interventions highlighted that a single policy change without community integration and continuous evaluation may not achieve the desired results and could exacerbate existing challenges.

## Implications for practice/policy

Findings from this scoping review highlight interventions that extend beyond clinical care, illustrated by the inclusion of a wide range of study designs, intervention types, and implementation levels. Healthcare systems should prioritize maintaining MOUD as the standard of care, as all three detoxification studies demonstrated concerning outcomes including 36% relapse rates. Anti-stigma training for both clinical providers and office staff is a critical implementation priority, with both educational interventions in our review demonstrating significant improvements in healthcare provider attitudes, bias, and compassion toward patients with OUD. Continuing education for anti-stigma practices should also be considered due to clinician and staff turnover. Most importantly, our findings reveal that effective prenatal OUD interventions require shifting from individual-level clinical case management to community-level system integration that formally coordinates healthcare, social services, and child welfare systems. Healthcare systems implementing these evidence-based approaches should focus on system integration, not merely service co-location, while simultaneously addressing the social determinants of health that individual clinical interventions cannot influence. These findings demonstrate that effective prenatal OUD interventions must move beyond traditional medical models to embrace comprehensive, community-integrated approaches that address the complex, multifaceted nature of maternal OUD and its impacts on families.

While this scoping review provides insights into the current landscape of prenatal OUD interventions and gaps, there are limitations. First, our review only included studies published in English and conducted in the United States, which may limit the generalizability of our findings to other contexts and countries. Second, while we intentionally excluded strictly pharmacological interventions, we may have omitted a review of studies that may have included implementation methods relevant to MOUD adherence and outcomes in pregnant individuals. However, 94% of our included studies reported participant MOUD use, allowing examination of intervention effectiveness within evidence-based pharmacotherapy approaches. Additionally, while our study included interventions conducted during the COVID-19 pandemic, our review did not systematically assess the impact of the pandemic on study implementation or outcomes, which may limit the generalizability of our findings to current practice. Lastly, our reliance on published studies may introduce publication bias, given that studies with negative or inconclusive findings are less likely to be published, which could skew the perceived effectiveness of interventions and limit the overall understanding of prenatal OUD intervention approaches.

## Conclusion

Pregnancy offers a unique and critical window for OUD interventions, as it enables regular access to healthcare services for treating chronic health conditions. However, our review demonstrates the critical importance of addressing prenatal OUD through integrated intervention strategies that encompass multiple socioecological levels and extend beyond access to MOUD. Our findings reveal that community-based approaches consistently outperformed individual-level interventions, demonstrating that effective interventions require shifting from clinical case management to system integration that formally coordinates healthcare, social services, and child welfare systems. Thus, effective interventions must adopt a holistic approach that addresses the broader social determinants of health and societal level barriers to recovery. Fostering collaboration among people with lived experiences, healthcare providers, policymakers, and community organizations is essential to empower pregnant individuals with OUD and promote healthier futures for their families.

## Data Availability

The original contributions presented in the study are included in the article/supplementary material. Further inquiries can be directed to the corresponding author.
